# Profiles of quality of life among patients using emergency departments for mental health reasons

**DOI:** 10.1186/s12955-023-02200-3

**Published:** 2023-10-26

**Authors:** Marie-Josée Fleury, Zhirong Cao, Guy Grenier, Francine Ferland

**Affiliations:** 1 Department of Psychiatry, McGill University, Douglas Mental Health University Institute Research Centre Montreal, Montreal, QC Canada; 2 Douglas Mental Health University Institute Research Centre Montreal, Montreal, QC Canada; 3 School of Social Work, Addiction Rehabilitation Center, Laval University, National Capital University Integrated Health and Social Services Center, Quebec City, QC Canada; 4 Douglas Hospital Research Centre, 6875 LaSalle Blvd, Montreal, QC H4H 1R3 Canada

**Keywords:** Quality of life, Mental disorders, Emergency department users, Profiles, Cluster analysis

## Abstract

**Background:**

This study identified profiles associated with quality of life (QoL) and sociodemographic and clinical characteristics of patients using emergency departments (ED) for mental health reasons and associated these profiles with patient service use.

**Methods:**

Recruited in four Quebec (Canada) ED networks, 299 patients with mental disorders (MD) were surveyed from March 1st, 2021, to May 13th, 2022. Data from medical records were collected and merged with survey data. Cluster analysis was conducted to identify QoL profiles, and comparison analyses used to assess differences between them.

**Results:**

Four QoL profiles were identified: (1) Unemployed or retired men with low QoL, education and household income, mostly having substance-related disorders and bad perceived mental/physical health conditions; (2) Men who are employed or students, have good QoL, high education and household income, the least personality disorders, and fair perceived mental/physical health conditions; (3) Women with low QoL, multiple mental health problems, and very bad perceived mental/physical health conditions; (4) Mostly women with very good QoL, serious MD, and very good perceived mental/physical health conditions.

**Conclusion:**

The profiles with the highest QoL (4 and 2) had better overall social characteristics and perceived their health conditions as superior. Profile 4 reported the highest level of satisfaction with services used. To improve QoL programs like permanent supportive housing, individual placement and support might be better implemented, and satisfaction with care more routinely assessed in response to patient needs – especially for Profiles 1 and 3, that show complex health and social conditions.

**Supplementary Information:**

The online version contains supplementary material available at 10.1186/s12955-023-02200-3.

## Background

Quality of life (QoL) is a key outcome in the planning and evaluation of health services, notably for patients with chronic illnesses or vulnerable populations – e.g., patients with mental disorders (MD), who often are frequent emergency department (ED) users, with 4 + ED visits/year [1–3]. According to the World Health Organization (WHO), QoL is a multidimensional concept that integrates various aspects of health and well-being in relation to the individual’s environment and beliefs [4]. QoL is strongly associated with the recovery movement [5] of patients achieving a satisfying life in spite of their illnesses or vulnerabilities [6]. Well-being, engagement in meaningful activities, low self-stigma, and the feeling of belonging to a community are characteristics of high QoL among patients with MD, including substance-related disorders (SRD) [7, 8]. QoL may also be used as a proxy for measuring the met needs of patients, access to care, or satisfaction with services [9, 10]. Patients using ED, and frequent ED users in particular, are often reported as having high unmet needs [11] and barriers to care [12], a consequence of usually insufficient access to and adequacy of outpatient care [13]. This suggests that high ED users could have low QoL. A better knowledge of QoL, sociodemographic and clinical profiles of ED users with MD, associated with outpatient service use and satisfaction, may sustain recommendations to improve health services for these vulnerable patients.

Though numerous studies investigate determinants of QoL among patients with MD [14–20], few have examined QoL profiles [21, 22]. In fact, we found no previous study investigating QoL profiles among ED users. While few published studies have identified ED user profiles among patients with MD, several have assessed determinants of ED use [23, 24] or high ED use [25–31] among such patients. A previous study [32] identified four QoL profiles among patients with MD; the profile with the best QoL was of older men with low education, serious MD, few important needs, and receiving a high level of help from health services; whereas the profile with the worst QoL included young women with co-occurring MD-SRD and important needs [32]. Yet another [33] reported four profiles of ED users with MD, with the one with the highest ED use also having the highest specialized service use among patients with multiple MD-SRD. Another recent study [34] showed three profiles of high ED users with MD: (1) 3-year recurrent very high ED users (10 + ED visits/year); (2) 2-year recurrent high ED users; (3) 1-year high ED users. Profiles differed in their severity of health conditions, intensity of outpatient service use, and risk of death, all of which were high in Profile 1, moderate in Profile 2, and low in Profile 3.

QoL studies usually focus on patients with specific MD: serious MD [14], bipolar disorders [15, 16], depressive disorders [17–19], borderline personality disorders [20], and SRD [35]. Studies found that QoL was mostly lower among women [15], patients with several MD [15, 20], general anxiety disorders [17], drug-related disorders [14], or more severe depressive symptoms [15, 16, 18, 20]. Conversely, individuals in a relationship [19], and who had better social integration [14], self-efficacity [16], schizophrenia [14], and few important needs [14] reported higher QoL. Based mainly on patient medical records, determinants of high ED use were mostly associated with being male [36, 37], younger [28, 29], poor [30], and diagnosed with schizophrenia [25, 31], SRD [31], or personality disorders [26, 27].

Investigating QoL profiles among ED users in association with their service use and level of satisfaction may provide complementary information that could enable health practitioners to better respond to the comprehensive needs of these patients, and decrease their high ED use. This study is original in that it investigates QoL among ED users based on both patient surveys and medical records, including a broad set of variables related to patient characteristics and service use data. Based on 299 patients who used different ED health networks for mental health (MH) reasons in Quebec (Canada), this study aimed to identify patient profiles based on their QoL, sociodemographic and clinical characteristics, and associated these profiles with patient service use. Identifying QoL profiles in this way might help decision makers deploy interventions that are more adapted to each profile, and provide more individualized treatment.

## Methods

### Study setting and data collection

The study was conducted in four Quebec (Canada) ED networks serving a population of about two million individuals – roughly one fourth of the province’s population. The first ED was in a large Montreal psychiatric hospital, while the second was also an ED in a psychiatric hospital but merged within a general hospital. Most patients had to go to the ED of the general hospital, from where they were referred to the psychiatric ED. The third was a psychiatric ED located in a large university-affiliated general hospital in Quebec City, where patients first went before being transferred to the psychiatric ED. The fourth ED, located in an outlying area of Montreal, was integrated within a general hospital where the staff included psychiatric clinicians.

**Fig. 1:**
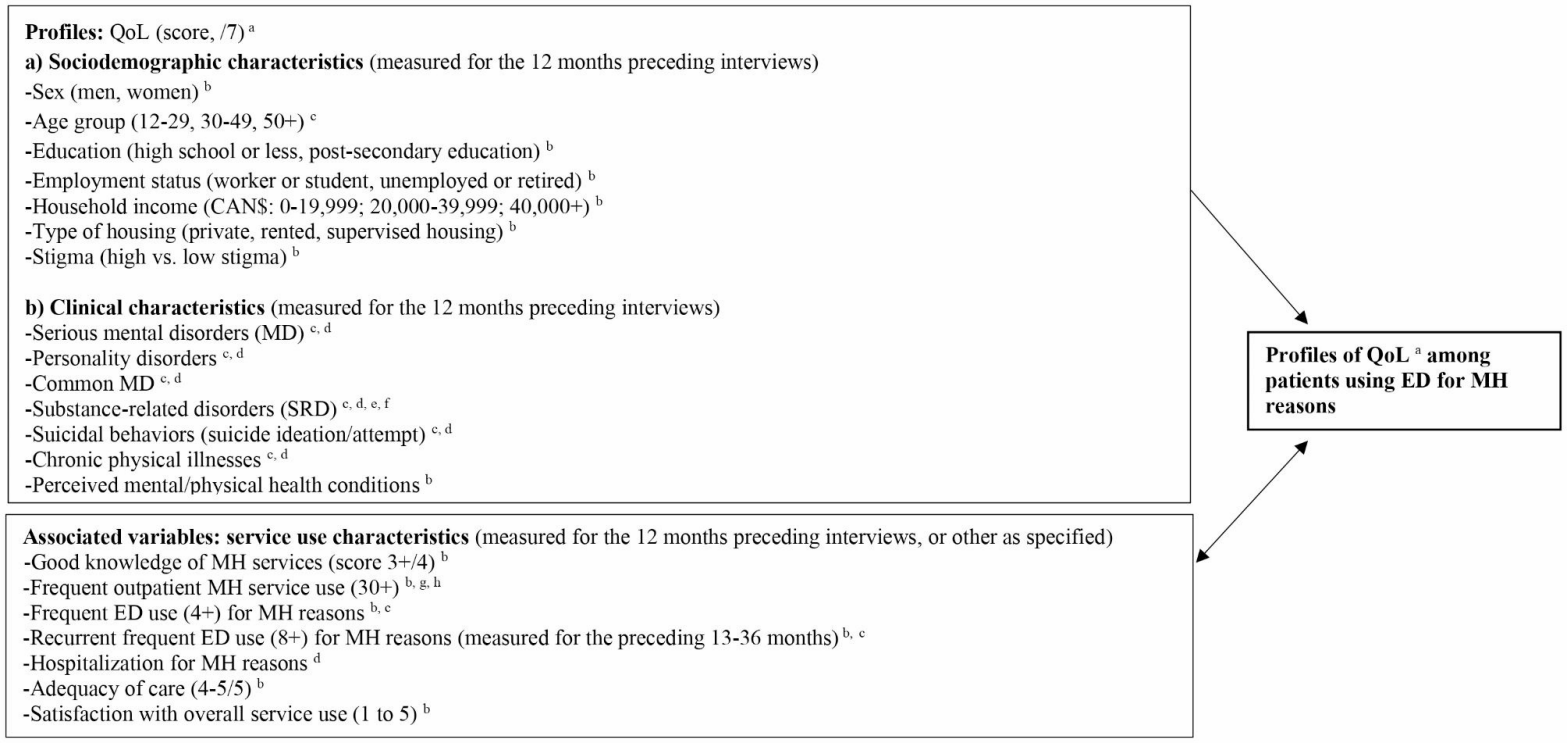
Analytical framework: Profiles of quality of life (QoL) among patients using emergency departments (ED) for mental health (MH) reasons (N = 299). ^a ^*Satisfaction with Life Domains Scale *(included in the survey); ^*b*^ Survey/questionnaire; ^c^*Banque de données communes des urgences *(BDCU, ED database); ^d^*Maintenance et exploitation des données pour l’étude de la clientèle hospitalière *(MED-ECHO, hospitalization database); ^e^*Alcohol Use Disorders Identification Test* (*AUDIT,* included in the survey); ^*f*^*Drug Abuse Screening Test-20 (DAST-20, *included in the survey); ^g^*Système d’information permettant la gestion de l’information clinique et administrative dans le domaine de la santé et des services sociaux *(I-CLSC, community health center database); ^h^ “*Psychiatric outpatient services used” *database. For the definitions of variables included in the study, see the Table 1 footnotes or the Methods section. Details for the diagnostic codes are presented in Appendix 1

Study participants had to be ED users for MH reasons, diagnosed with MD including SRD, 18 + years old, able to complete a survey, French or English speakers, and had to grant the research team access to their medical records within the targeted networks. Recruitment was conducted randomly from a list of 1,751 ED users with MD, identified as such in the medical records filled by ED clinicians from these networks. The coordinates of the first 450 patients that ED clinicians identified as interested in participating in the study were referred to the research team as potential study participants. The research coordinator then contacted these individuals, asking them to take part in a survey conducted by trained interviewers, with close monitoring from the research team. The surveys were conducted by telephone, due to restrictions regarding face-to-face contact during the COVID-19 pandemic. They were administered between March 1st, 2021 and May 13th, 2022, each interview lasting around 45 min. As the surveys went on, medical records data were collected for the 12 months preceding patient interviews, except for recurrent frequent ED use, which was measured for the 2 years preceding the 12 months of ED use targeted in the interviews. Medical records included information about ED use (BDCU database; e.g., date, reason for visit – *Banque de données communes des urgences*) [38] and hospitalization for MH reasons (MED-ECHO database; e.g., dates of admission/discharge, duration of inpatient stay – *Maintenance et exploitation des données pour l’étude de la clientele hospitalière*) [39], psychiatric outpatient services used (hospital database; e.g., date, type of services received), and psychosocial MH services received from community healthcare centers (I-CLSC database; e.g., date, type of services received – *Système d’information sur la clientèle et les services des centres locaux de services communautaires*) [40]. Patient diagnoses were based on the BDCU and MED-ECHO databases, framed by the International Classification of Diseases, Tenth Revision (**Appendix 1**). All databases included information on patient service use (e.g., frequency) exclusively within the ED networks. A steering committee including ED clinicians validated the survey data to be collected, including data on integrated service use outside the ED networks and data on services other than those in network hospitals and community healthcare centers (e.g., medical clinics, community-based organizations), which completed the information taken from medical records. The variables extracted from the survey data and individual medical records are presented in a conceptual framework (Fig. 1) including the standardized scales used. Participation in the study was voluntary. Patients provided oral consent and received a modest financial compensation. The ethics review board of the Douglas Mental Health University Institute approved the multi-site protocol.

### Study variables

The dependent variable, QoL, was assessed using the Satisfaction with Life Domains Scale, a 20-item standardized questionnaire based on a 7-point scale, with higher scores indicating better QoL [41, 42]. Apart from QoL, patient profiles included sociodemographic and clinical variables, which were then associated with service use to differentiate patients with MD using ED.

Sociodemographic characteristics included: sex, age group, education (e.g., high school or less), employment status (e.g., worker, unemployed), household income (CAN$), type of housing (e.g., supervised), and level of stigma. In line with the Canadian Community Health Survey [43] and previous studies that have assessed stigma using a one-item question [44, 45], stigma was measured using the following question, with responses on a 5-point scale ranging from “totally disagree” to “totally agree”: *Most people in my community treat a person with a MD or SRD in the same manner as they would any other person*. Responses of “totally disagree” and “somewhat disagree” represented high stigma.

Clinical characteristics included: MD, suicidal behaviors (suicide ideation or attempt), good perceived mental/physical conditions, and chronic physical illnesses (e.g.: heart diseases, diabetes). MD included serious MD (schizophrenia spectrum and other psychotic disorders, bipolar disorders), personality disorders, common MD (anxiety, depressive and adjustment disorders, attention deficit/hyperactivity disorder), and SRD integrating alcohol- and drug-related disorders (use, induced, intoxication and withdrawal). SRD were measured using medical records. Considering that SRD are often under-diagnosed [46], the Alcohol Use Disorders Identification Test [47] and the Drug Abuse Screening Test-20 [48] were also used. The list of chronic physical illnesses was based on an adapted version integrating both the Charlson and Elixhauser Comorbidity indexes [49]. Perceived mental/physical health conditions were measured with two questions based on the Canadian Community Health Survey [43]. The questions (*How do you see your MH? How do you see your physical health?*) were on a 10-point scale ranging from poor to excellent, with 7 + representing good perceived mental/physical health conditions.

Service use included: knowledge of MH services; frequent outpatient MH service use; frequent ED use, recurrent frequent ED use, and hospitalization for MH reasons (yes/no); adequacy of care; and satisfaction with overall service use. Knowledge of MH services was measured with the following question: *How do you evaluate your knowledge of MH or addiction services?* This question on a 4-point scale ranged from poor or fair to excellent, with 3 + considered very good to excellent knowledge. Outpatient MH services included consultations with general practitioners (family doctors or GP from walk-in clinics), in community healthcare centers providing mostly psychosocial services, and with psychologists in private practice. They also included services from psychiatrists, most of whom (about 90% of Quebec psychiatrists  [50]) worked in hospital multidisciplinary teams, assertive community treatment and intensive case management programs, addiction rehabilitation centers, and community-based organizations (e.g., crisis or suicide prevention centers). A benchmark of 30 + services/year was established for frequent outpatient service use, representing roughly 2.5 + services/month, or 3 + over a 10-month period. An ED use frequency of 4 + visits/year is a standard definition used in Canadian studies [51, 52]. Recurrent frequent ED use was defined as 8 + visits over the 2 years preceding the 12 months considered in the surveys. Previous studies have shown that frequent ED users often use ED for several consecutive years, thus contributing to the “revolving door syndrome” [53, 54]. Adequacy of care was measured with a question with a 5-point response scale, from the Canadian Community Health Survey [43]: *Do services outside of ED respond to your needs?* Answers included “(1) I totally disagree”, “(4) I agree”, and “(5) I totally agree”, with good adequacy of care corresponding to answers 4 and 5. The service satisfaction variable considered for each service used by patients was measured on a 5-point scale ranging from “(1) Not at all satisfied” to “(5) Totally satisfied”. The mean level of satisfaction with services used per patient was also considered, with higher scores indicating greater satisfaction.

### Analysis

Missing values (less than 1%) were randomly distributed and imputed by mean and mode [55]. Descriptive analyses were produced by frequency distributions for categorical variables, and mean values with standard deviations (SD.) for continuous variables. Cluster analysis was conducted using the k-means group algorithm with Gower dissimilarity coefficient based on QoL, sociodemographic and clinical characteristics. Several k-means solutions with different numbers (3–7) of profiles were computed [56]. The four-group solution had the largest Calinski–Harabasz pseudo-F value [57], indicating that it was the most distinct compared to the other models. Comparison analyses were conducted to assess statistical differences between profiles, using Chi-square or Fisher’s exact tests for categorical variables, and T-tests for continuous variables. Analyses were performed using Stata 17 [58].

## Results

Of the 450 ED users referred to the research team, 50 could not be reached and 300 accepted to participate in the study (75% response rate). One patient was withdrawn as data collection was completed twice for this patient in two ED networks. The mean QoL score for the 299 patients in the final sample was 4.55/7 (Table 1). Most patients were 30 + years old (69%), 55% were women, 57% unemployed or retired, 57% had some post-secondary education, 22% lived in supervised housing, 50% had high perceived stigma, and 47% had a household income of less than CAN$20,000/year. Most (59%) reported having SRD, 57% common MD, 45% serious MD, 42% personality disorders, 54% suicidal behaviors, 45% chronic physical illnesses, and 32% perceived their mental/physical health conditions as good (7+/10). As for service use, 54% reported having good knowledge of MH services, 51% showed frequent outpatient service use (30 + times/year), 61% frequent ED use, 39% recurrent frequent ED use, 56% were hospitalized, and 63% had received adequate care. The mean score for overall service use satisfaction was 3.96/5.0.

**Table 1:** Characteristics of patients (N = 299)

	Mean	SD.
**Quality of life** (QoL, /7) ^a^	4.55	1.06
	** N**	**%**
**Sociodemographic characteristics** (measured for the 12 months preceding interviews)		
Sex		
Women	165	55.18
Men	134	44.82
Age		
16–29 years	92	30.77
30–49 years	117	39.13
50 + years	90	30.10
Education		
High school or less	130	43.48
Post-secondary education	169	56.52
Employment status		
Unemployed or retired ^b^	169	56.52
Worker or student	130	43.48
Household income (Canadian dollars/year)		
CAD$ 0–19,999	141	47.16
CAD$ 20–39,999	89	29.77
CAD$ 40,000+	69	23.07
Type of housing		
Private	60	20.07
Rented	173	57.86
Supervised ^c^	66	22.07
Stigma ^d^		
High	149	49.83
Low	150	50.17
**Clinical characteristics** (measured for the 12 months preceding interviews)		
Serious mental disorders (MD) ^e^	133	44.48
Personality disorders ^e^	127	42.47
Common MD ^e^	169	56.52
Substance-related disorders (SRD) ^e^	175	58.53
Suicidal behaviors (suicide ideation/attempt)	161	53.85
Good perceived mental/physical health conditions (score 7+/10)	95	31.77
Chronic physical illnesses ^f^	136	45.48
**Service use characteristics** (measured for the 12 months preceding interviews, or other as specified)		
Very good to excellent knowledge of mental health (MH) services (including addiction services, score 3+/4)	161	53.85
Frequent outpatient MH service use (30+) ^g^	153	51.17
Frequent emergency department (ED) use (4+) for MH reasons	182	60.86
Recurrent frequent ED use (8+) (measured for the preceding 13–36 months) for MH reasons	117	39.13
Hospitalization for MH reasons	168	56.19
Adequacy of care (4–5/5) ^h^	187	62.54
	**Mean**	**SD.**
Satisfaction with global service use ^i^	3.96	0.76

^a^ QoL: score rating from 1 to 7, higher score = better quality of life.

^b^ The sample was too small to separate unemployed from retired.

^c^ Supervised housing included different types of housing such as group homes, residential care, and supported apartments.

^d^ Patients were asked this question in the survey: “Most people in my community treat a person with a MD or a SRD in the same manner as they would treat any other person”. Please tell me if you: (1) totally disagree, (2) somewhat disagree, (3) somewhat agree, (4) agree, or (5) totally agree with this statement. High stigma related to responses 1 and 2, medium stigma to response 3, and low stigma to responses 4 and 5. This question is based on the Canadian community health survey (CCHS).

^e^ Patients may have more than one MD. Based on medical records, serious MD included: schizophrenia spectrum and other psychotic disorders, and bipolar disorders; common MD: anxiety, depressive, and adjustment disorders, and attention deficit/hyperactivity disorder; SRD: alcohol- or drug-related disorders (use, induced, intoxication and withdrawal). Personality disorders and SRD were also based on medical records, but SRD were also measured with standardized scales (see the Methods section, and Appendix 1).

^f^ Based on medical records, chronic physical illnesses included: chronic pulmonary disease, cardiac arrhythmia, tumor with or without metastasis, renal disease, fluid electrolyte disorder, myocardial infarction, congestive heart failure, metastatic cancer, dementia, stroke, neurological disorder, liver disease, pulmonary circulation disorder, coagulopathy, weight loss, paralysis, AIDS/HIV (see Appendix 1).

^g^ Outpatient services included use of family doctors, general practitioners (GP) from walk-in clinics, community healthcare centers (mostly psychosocial services), and psychologists in private practice, psychiatric services including assertive community treatment or intensive case management programs, addiction rehabilitation centers, and community-based organizations (e.g., crisis or suicide prevention centers).

^h^ Patients were asked if “services outside of the ED respond to their needs”. They could respond: (1) I totally disagree, (2) I somewhat disagree, (3) I somewhat agree, (4) I agree, and (5) I totally agree. High adequacy of care corresponded to answers 4 and 5.

^i^ For each service used, patients were asked to express their level of satisfaction, measured on a 5-point scale. The mean level of satisfaction with service use per patient was considered. A higher score indicated greater satisfaction with services.

Cluster analysis identified four groups with quite different QoL scores and sample sizes. The superscript numbers in Table 2 indicate significant differences between profiles, as reported below. Accounting for 25% of the sample, Profile 1 had the lowest QoL score (3.99/7). All Profile 1 patients were men, differing as such from Profiles 3 and 4 which included few men (0% and 33%, respectively). Profile 1 had the most patients who were unemployed or retired (82%) and showed the lowest household income of all profiles, with 73% reporting earnings of less than CAN$20,000/year. Profile 1 also had more patients with high school education or less compared to Profile 2 (27% vs. 6%), and more living in supervised housing (55% vs. 31%). Profile 1 patients were more affected by SRD than those of Profile 4 (69% vs. 51%), but less affected than Profile 3 by personality disorders (31% vs. 57%) and common MD (47% vs. 67%). Only 11% of Profile 1 patients perceived having good mental/physical health conditions, many fewer than in Profiles 4 and 2 (100% and 31%, respectively), but still more than Profile 3 (0%). Profile 1 was labeled: “Unemployed or retired men with low QoL, education and household income, mostly having SRD and bad perceived mental/physical health conditions”.

**Table 2:** Characteristics and comparisons between profiles of the four-group model (N = 299)

	Profile 1*	Profile 2*	Profile 3*	Profile 4*
**Group size: N (%)**	74 (24.75%)	35 (11.71%)	114 (38.13%)	76 (25.42%)
	**Mean (SD.)**	**Mean (SD.)**	**Mean (SD.)**	**Mean (SD.)**
**Quality of life** (QoL, /7) ^a^	3.99 (1.03)^2,3,4^	4.99 (0.89)^1,3^	4.31 (0.94)^1,2,4^	5.25 (0.85)^1,3^
	**%**	**%**	**%**	**%**
**Sociodemographic characteristics** (measured for the past 12 months preceding interviews)				
Sex				
Women	0^3,4^	0^3,4^	100^1,2,4^	67.11^1,2,3^
Men	100	100	0	32.89
Age				
16–29 years	27.02	20.00	36.84	30.26
30–49 years	36.49	51.43	34.21	43.42
50 + years	36.49	28.57	28.95	26.32
Education				
High school or less	54.05^2^	31.43^1^	40.35	43.42
Post-secondary education	45.95	68.57	59.65	56.58
Employment status				
Unemployed or retired ^b^	82.43^2,3,4^	28.57^1,3^	57.02^1,2^	43.42^1^
Worker or student	17.57	71.43	42.98	56.58
Household income (Canadian dollars)				
CAN$0–19,999	72.97^2,3,4^	0^1,3,4^	50.88^1,2^	38.16^1,2^
CAN$20–39,999	27.03	0	29.82	46.05
CAN$40,000+	0	100	19.30	15.79
Type of housing				
Private	13.51^2^	25.71^1,3^	23.68^2^	18.42
Rented	59.46	68.57	50.88	61.84
Supervised ^c^	27.03	5.72	25.44	19.74
Stigma ^d^				
High	51.35	42.86	54.39	44.74
Low	48.65	57.14	45.61	55.26
**Clinical characteristics** (measured for the past 12 months preceding interviews)				
Serious mental disorders (MD) ^e^	41.89	45.71	38.6^4^	55.26^3^
Personality disorders ^e^	31.08^3^	20.00^3,4^	57.02^1,2,4^	42.11^2,3^
Common MD ^e^	47.30^3^	48.57	66.67^1^	53.95
Substance-related disorders (SRD) ^e^	68.92^4^	62.86	55.26	51.32^1^
Suicidal behaviors (suicide ideation/attempt)	54.05	40.00^3^	61.40^2^	48.68
Good perceived mental/physical health conditions (7+/10)	10.81^2,3,4^	31.43^1,3,4^	0^1,2,4^	100^1,2,3^
Chronic physical illnesses ^f^	41.89	42.86	48.25	46.05

Superscript numbers indicate significant differences between profiles at p < 0.05

*Profile 1: Unemployed or retired men with low QoL, education and household income, mostly having SRD, and bad perceived mental/physical health conditions

*Profile 2: Men who are employed or students, with good QoL, high education and household income, the least personality disorders, and fair perceived mental/physical health conditions

*Profile 3: Women with low QoL, multiple mental health problems, and very bad perceived mental/physical conditions

*Profile 4: Mostly women with very good QoL, serious MD, and very good perceived mental/physical health conditions

^a^ See note ^a^ below Table 1

^b^ See note ^b^ below Table 1

^c^ See note ^c^ below Table 1

^d^ See note ^d^ below Table 1

^e^ See note ^e^ below Table 1

^f^ See note ^f^ below Table 1

Accounting for 12% of the sample, Profile 2 had the highest QoL score along with Profile 4 (4.99 vs. 5.25/7, so not significantly different). Like Profile 1, Profile 2 only included men. Out of the four profiles, Profile 2 patients showed the highest household income, with earnings of CAN$40,000+/year. They were workers or students in higher numbers (71%) than in Profiles 1 and 3 (18%, 43%), and more of them lived in private or rented housing (94%) compared to Profiles 1 and 3 (73%, 75%). More Profile 2 patients had a post-secondary education compared to Profile 1 (69% vs. 46%). They were less affected by personality disorders (20%) than those of Profiles 3 and 4 (57%, 42%), and showed less suicidal behaviors than Profile 3 (40% vs. 61%). Profile 2 ranked second in perceived mental/physical health conditions, with 31% perceiving good conditions. Profile 2 was labeled: “Men who are employed or students, have good QoL, high education and household income, the least personality disorders, and fair perceived mental/physical health conditions”.

Accounting for 38% of the sample, Profile 3 had the lowest QoL score (3.99/7) after Profile 1. All Profile 3 patients were women, and none perceived having good mental/physical health conditions. Most (51%) reported a household income below CAN$20,000/year, with 57% of them being unemployed or retired – both figures lower here than in Profile 1 (73%, 82%), but higher than Profile 2 (0%, 29%). Profile 3 patients were more likely to live in supervised housing than those in Profile 2 (25% vs. 6%). Most were affected by personality disorders (57%), more had common MD than in Profile 1 (67% vs. 47%) and suicidal behaviors than in Profile 2 (61% vs. 40%), but fewer had serious MD compared to Profile 4 (39% vs. 55%). Profile 3 was labeled: “Women with low QoL, multiple MH problems, and very bad perceived mental/physical health conditions”.

Accounting for 25% of the sample, Profile 4 had the highest QoL score (5.25/7), although comparable to Profile 2 (4.99). Most were women (67%), all perceiving their mental/physical health conditions as good. Of all Profile 4 patients, 38% reported a household income of less than CAN$20,000/year; by contrast 51% of Profile 3, 73% of Profile 1, and 0% of Profile 2 were at that income level. With 43% unemployed or retired, Profile 4 patients differed from those of Profile 1, where 82% were working or studying. Profile 4 included more patients with personality disorders (42%) than in Profile 2 (20%), but fewer than in Profile 3 (57%). Profile 4 also had more patients with serious MD than Profile 3 (55% vs. 39%), but fewer with SRD than in Profile 1 (51% vs. 69%). Profile 4 was labeled: “Mostly women with very good QoL, serious MD, and very good perceived mental/physical health conditions”.

Patient profiles also differed regarding service use (Table 3). Profile 4 patients showed the highest satisfaction with their overall service use (4.27/5), had better knowledge of MH services (66%) than Profiles 1 and 2 (both 46%), and reported higher adequacy of care than Profile 3 (71% vs. 56%). Profile 3 patients, on the other hand, accounted for more frequent ED use and outpatient service use (70%, 60%) than Profiles 1 (69%, 46%) and 4 (72%, 55%). Recurrent frequent ED use was higher in Profile 3 (49%) than in Profiles 2 and 4 (26%, 30%).

**Table 3:** Associations between profiles and service use (N = 299)

	Profile 1*	Profile 2*	Profile 3*	Profile 4*
**Group size: N (%)**	74 (24.75%)	35 (11.71%)	114 (38.13%)	76 (25.42%)
	**%**	**%**	**%**	**%**
**Service use characteristics** (measured within the past 12 months before interviews, or other as specified)				
Very good to excellent knowledge of mental health (MH) services (including addiction services, 3+/4)	45.95^4^	45.71^4^	53.51	65.79^1,2^
Frequent outpatient MH service use (30+) ^a^	45.95^3^	45.71	60.53^1,4^	44.74^3^
Frequent emergency department (ED) use (4+) for MH reasons	51.35^3^	62.86	70.18^1,4^	55.26^3^
Recurrent frequent ED use (8+) (measured in the past 13–36 months) for MH reasons	39.19	25.71^3^	49.12^2,4^	30.26^3^
Hospitalization for MH reasons	59.46	54.29	60.52	47.37
Adequacy of care (4–5/5) ^b^	59.46	71.43	56.14^4^	71.05^3^
	**Mean (SD.)**	**Mean (SD.)**	**Mean (SD.)**	**Mean (SD.)**
Satisfaction with overall service use ^c^	3.92 (0.79)^4^	3.95 (0.85)^4^	3.80 (0.76)^4^	4.27 (0.59)^1,2,3^

Superscript numbers indicate significant differences between profiles at p < 0.05

*See notes *Profiles below Table 2

^a^ See note ^g^ below Table 1

^b^ See note ^h^ below Table 1

^c^ See note ^i^ below Table 1

## Discussion

This study identified four QoL patient profiles among ED users based on their sociodemographic and clinical characteristics, and associated these profiles with their service use. The study patients’ mean QoL score was relatively low (4.55/7), which could be explained by their overall high prevalence of social and health problems. Roughly half were unemployed or retired, had low household income, high perceived stigma, serious MD, SRD, personality disorders, suicidal behaviors or co-occurring chronic physical illnesses. Over 20% of them were living in supervised housing. Overall, study patients were also frequent service users, with most showing frequent ED use: over 30% reported frequent ED use for 3 consecutive years, and more than 50% had been hospitalized for MH reasons.

Profiles 2 and 4 had the best QoL, with two-thirds of the latter composed of women and all of the former consisting of men; although it should be noted that Profile 2 accounted for only 12% of the sample. All Profile 4 patients perceived their mental/physical health conditions as good, as did a third of those in Profile 2, which ranked second in this respect. Profile 4 included the most patients with serious MD, followed by Profile 2, which had the least patients with personality disorders. Self-perceived well-being is a key predictor of QoL [8] and a positive health outcome [59]. Studies have shown that patients with serious MD have better QoL than those with other MD [60] or low income [61]. The recovery movement may have helped patients with serious MD to lead a good life despite their chronic illnesses. These patients may also have entertained fewer expectations, especially if they received the help they needed. Like patients with personality disorders, they are known to have low QoL [62]. Profile 2 had the best social conditions, with Profile 4 a close second. These two profiles had the most patients working or studying, those with more household income and fewer (especially Profile 2) living in supervised housing. Indeed, previous studies have found that having a job increases self-esteem [63], reduces the need for treatment [63], and has a positive impact on health-related QoL [64], especially among patients with serious MD. Study patients usually preferred to live autonomously whenever possible [65], which may explain higher QoL in Profile 2 and 4 patients, who lived mainly in private or rented housing. Profile 4 patients were the most satisfied with services received, and, along with Profile 2, reported the highest adequacy of care. Profile 4 patients also had the best knowledge of MH services, which may have helped them get the help they needed. Higher appreciation of services in Profiles 4 and 2 may have contributed to their better overall situation and QoL compared to Profiles 1 and 3.

Profile 1 patients, who were all men, had the lowest QoL, the most disadvantaged social characteristics, and were the most affected by SRD, with fewer perceiving their mental/physical conditions as good. While half of patients in Profile 3 reported recurrent frequent ED use, those in Profile 1 also scored high in this regard, with most being unemployed and having low household income (<$20,000). The negative associations between poverty, unemployment, poor perceived health conditions and QoL have often been reported [66, 67], and thus, contrary to the men in Profile 2, about a third of Profile 1 patients were living in supervised housing, which may have contributed to their lower QoL scores. Previous studies have shown that QoL may be lower among patients living in such settings, compared to those living autonomously [65]. Previous studies also found poor QoL among patients with SRD [35, 68], which was associated with loss of a job or relationship [69, 70], higher morbidity [71], and affected many more men than women [72]. Men and patients with SRD are known to be frequent ED users [73, 74]. Compared with women, men tend to use health services as a last resort [75]. Patients like those in Profile 1, who make high use of ED over several consecutive years, are also known to be a vulnerable group. This pattern, compounded by the cumulative impact of deprivation and low health perception, may explain why Profile 1 showed the worst QoL.

Profile 3 was second worst in QoL, with patients sharing the same disadvantaged social characteristics found in Profile 1 (e.g., unemployment, low income, living in supervised housing), with the difference that all Profile 3 patients were women. They were the most affected by personality disorders, common MD, and suicidal behaviors, and were the most frequent service users, none of whom perceived their mental/physical conditions as good. In our study as in the existing literature, patients with personality disorders, and particularly borderline personality disorders, are found to have great difficulty maintaining jobs and relationships [76, 77], more negative perceptions of their physical mental/conditions [78, 79], and more likely to be high service users [80]. The greater prevalence of common MD compared to Profile 1, and of suicidal behaviors compared to Profile 2, may also be related to personality disorders, as those are often associated [81]; this prevalence may explain why Profile 3 accounted for nearly 40% of the sample. Suicidal behaviors, for their part, have quite logically been negatively associated with QoL [82]. Considering their numerous social and health issues, it is no surprise that Profile 3 patients are known to be high ED [83] and outpatient care users [80]. These characteristics may explain why they had the second worst QoL after Profile 1.

### Limitations

This study has some limitations. First, patients were recruited from ED with psychiatric care in large urban areas, operating within a public healthcare system, so findings may not be generalizable to other types of ED, territories, or contexts. Second, only overall QoL was measured. If some aspects of QoL (e.g., health, living situation) had been measured separately, they might have been more strongly associated with specific patient characteristics and patterns of service use. Third, as the study was cross-sectional, the causality between associations could not be established. Fourth, outpatient MH care was assigned only to one variable. Finer details about services could have enabled us to further distinguish quality of care between profiles. Finally, it was impossible to test how the COVID-19 pandemic may have affected the QoL of ED users. Data collection for the study did not however occur during the first waves of the COVID-19 pandemic, which had the worse impact on ED users [84].

## Conclusion

This study was original in identifying four distinct QoL profiles among patients who were high ED users: Profiles 4 and 2 reported high QoL; Profile 1, low QoL; and Profile 3, moderate QoL. Men and women were always contrasting in high QoL as opposed to low/moderate QoL profiles, with Profiles 1 and 2 including only men, Profile 3 only women, and two-thirds of Profile 4 comprised of women. The study showed that profiles with the highest QoL (4 and 2) also had better social characteristics and perceived health conditions. And while Profile 4 had the best QoL (although not significantly higher than Profile 2), it also registered the highest level of satisfaction with services received. Consequently, interventions aimed at improving QoL may be recommended to enhance the social conditions of patients, especially employment and household income. Programs like permanent supportive housing, and individual placement and support might be suggested, especially for Profiles 1 and 3. In their practices, clinicians could be more attentive to patients, and integrate more individualized treatments to better respond to their patients’ perceived needs. The recovery approach could be promoted more, and alliances made with patients for implementing more adequate care. Satisfaction with care, found to be a promising measure associated with high QoL, might be more routinely assessed within the healthcare system to benefit patients.

### Electronic supplementary material

Below is the link to the electronic supplementary material.

Supplementary Material 1

## Data Availability

In accordance with the applicable ethics regulations for the province of Quebec, the principal investigator is responsible for keeping data confidential.

## Abbreviations

ED: Emergency department
MD: Mental disorders
MH: Mental Health
QoL: Quality of life
SD: Standard deviation
SRD: Substance-related disorders
WHO: World Health Organization

## References

[1] Caron J, Mercier C, Diaz P, Martin A (2005). Socio-demographic and clinical predictors of quality of life in patients with schizophrenia or schizo-affective disorder. Psychiatry Res.

[2] Ritsner M, Gibel A, Ratner Y (2006). Determinants of changes in perceived quality of life in the course of schizophrenia. Qual Life Res.

[3] Boyer L, Millier A, Perthame E, Aballea S, Auquier P, Toumi M (2013). Quality of life is predictive of relapse in schizophrenia. BMC Psychiatry.

[4] World Heath Organization (WHO). The World Health Organization quality of life assessment (WHOQOL) (1995). Position paper from the World Health Organization. Soc Sci Med.

[5] Anthony WA (1993). Recovery from the mental Illness: the guiding vision of the mental health service system in the 1990s. Psychiatr Rehabil J.

[6] Slade M, Bird V, Chandler R, Clarke E, Craig T, Larsen J, Lawrence V, le Boutillier C, Macpherson R, McCrone P (2017). REFOCUS: developping a Recovery Focus in Mental Health Services in England.

[7] Connell J, Brazier J, O’Cathain A, Lloyd-Jones M, Paisley S (2012). Quality of life of people with mental health problems: a synthesis of qualitative research. Health Qual Life Outcomes.

[8] Connell J, O’Cathain A, Brazier J (2014). Measuring quality of life in mental health: are we asking the right questions?. Soc Sci Med.

[9] Flanagan S, Damery S, Combes G (2017). The effectiveness of integrated care interventions in improving patient quality of life (QoL) for patients with chronic conditions. An overview of the systematic review evidence. Health Qual Life Outcomes.

[10] Kamenov K, Twomey C, Cabello M, Prina AM, Ayuso-Mateos JL (2017). The efficacy of psychotherapy, pharmacotherapy and their combination on functioning and quality of life in depression: a meta-analysis. Psychol Med.

[11] Vandyk AD, Harrison MB, VanDenKerkhof EG, Graham ID, Ross-White A (2013). Frequent emergency department use by individuals seeking mental healthcare: a systematic search and review. Arch Psychiatr Nurs.

[12] Walker ER, Fukuda J, McMonigle M, Nguyen J, Druss BG (2021). A qualitative study of barriers and facilitators to transitions from the Emergency Department to Outpatient Mental Health Care. Psychiatr Serv.

[13] Nesper AC, Morris BA, Scher LM, Holmes JF (2016). Effect of Decreasing County Mental Health Services on the Emergency Department. Ann Emerg Med.

[14] Fleury MJ, Grenier G, Bamvita JM, Tremblay J, Schmitz N, Caron J (2013). Predictors of quality of life in a longitudinal study of users with severe mental disorders. Health Qual Life Outcomes.

[15] Sylvia LG, Montana RE, Deckersbach T, Thase ME, Tohen M, Reilly-Harrington N, McInnis MG, Kocsis JH, Bowden C, Calabrese J (2017). Poor quality of life and functioning in bipolar disorder. Int J Bipolar Disord.

[16] Abraham KM, Miller CJ, Birgenheir DG, Lai Z, Kilbourne AM (2014). Self-efficacy and quality of life among people with bipolar disorder. J Nerv Ment Dis.

[17] Zhou Y, Cao Z, Yang M, Xi X, Guo V, Fang M, Cheng L, Du Y. Comorbid generalized anxiety disorder and its association with quality of life in patients with major depressive disorder. Sci Rep 2017, 7. 10.1038/srep40511 PMC5241829 28098176

[18] Sivertsen H, Bjorklof GH, Engedal K, Selbaek G, Helvik AS (2015). Depression and Quality of Life in older persons: a review. Dement Geriatr Cogn Disord.

[19] Dionisie V, Puiu M, Manea M, Pacearca IA. Predictors of changes in quality of life of patients with major depressive Disorder—A prospective naturalistic 3-Month Follow-Up study. J Clin Med 2023, 12. 10.3390/jcm12144628 PMC10380991 37510745

[20] Stefanatou P, Xenaki LA, Konstantakopoulos G, Papaiakovou A, Ralli I, Berk AD, Katopodi DS, Pantagoutsou AD, Charitaki A, Ginieri-Coccossis M et al. Psychopathological determinants of quality of life in people with Borderline personality disorder. J Clin Med 2022, 12. 10.3390/jcm12010030 PMC9820836 36614831

[21] De Maeyer J, van Nieuwenhuizen C, Bongers IL, Broekaert E, Vanderplasschen W (2013). Profiles of quality of life in opiate-dependent individuals after starting Methadone treatment: a latent class analysis. Int J Drug Policy.

[22] Liao Z, Allott K, Anderson JFI, Killackey E, Cotton SM (2022). Quality of life in first episode psychosis: a cluster analytic approach. Qual Life Res.

[23] Ayangbayi T, Okunade A, Karakus M, Nianogo T (2017). Characteristics of Hospital Emergency Room visits for Mental and Substance Use disorders. Psychiatr Serv.

[24] Saini P, McIntyre J, Corcoran R, Daras K, Giebel C, Fuller E, Shelton J, Wilson T, Comerford T, Nathan R, Gabbay M (2020). Predictors of emergency department and GP use among patients with mental health conditions: a public health survey. Br J Gen Pract.

[25] Kaltsidis G, Bamvita JM, Grenier G, Fleury MJ. Predictors of Frequent Emergency Department Utilization for Mental Health Reasons. J Behav Health Serv Res 2020. 10.1007/s11414-020-09695-4 32185614

[26] Richard-Lepouriel H, Weber K, Baertschi M, DiGiorgio S, Sarasin F, Canuto A (2015). Predictors of recurrent use of psychiatric emergency services. Psychiatr Serv.

[27] Chang G, Weiss AP, Orav EJ, Rauch SL (2014). Predictors of frequent emergency department use among patients with psychiatric Illness. Gen Hosp Psychiatry.

[28] Boyer L, Dassa D, Belzeaux R, Henry JM, Samuelian JC, Baumstarck-Barrau K, Lancon C (2011). Frequent visits to a French psychiatric emergency service: diagnostic variability in psychotic disorders. Psychiatr Serv.

[29] Sirotich F, Durbin A, Durbin J (2016). Examining the need profiles of patients with multiple emergency department visits for mental health reasons: a cross-sectional study. Soc Psychiatry Psychiatr Epidemiol.

[30] Moulin A, Evans EJ, Xing G, Melnikow J (2018). Substance Use, Homelessness, Mental Illness and Medicaid Coverage: a set-up for high Emergency Department utilization. West J Emerg Med.

[31] Vu F, Daeppen JB, Hugli O, Iglesias K, Stucki S, Paroz S, Canepa Allen M, Bodenmann P (2015). Screening of mental health and substance users in frequent users of a general Swiss emergency department. BMC Emerg Med.

[32] Fleury MJ, Grenier G, Bamvita JM (2015). Predictive typology of subjective quality of life among participants with severe mental disorders after a five-year follow-up: a longitudinal two-step cluster analysis. Health Qual Life Outcomes.

[33] Fleury MJ, Grenier G, Bamvita JM, Ferland F (2020). Typology of patients who use emergency departments for mental and substance use disorders. BJPsych Open.

[34] Armoon B, Cao Z, Grenier G, Meng X, Fleury MJ (2022). Profiles of high emergency department users with mental disorders. Am J Emerg Med.

[35] Armoon B, Fleury MJ, Bayat AH, Bayani A, Mohammadi R, Griffiths MD (2022). Quality of life and its correlated factors among patients with substance use disorders: a systematic review and meta-analysis. Arch Public Health.

[36] Fleury MJ, Rochette L, Grenier G, Huynh C, Vasiliadis HM, Pelletier E, Lesage A (2019). Factors associated with emergency department use for mental health reasons among low, moderate and high users. Gen Hosp Psychiatry.

[37] Schmidt M (2018). Frequent visitors at the psychiatric emergency room - A literature review. Psychiatr Q.

[38] Régie de l’assurance maladie du Québec. Banque des données communes ds urgences (BDCU). https://www.ramqgouvqcca/fr/media/8441.

[39] Ministère de la Santé et des Services Sociaux (2023). Cadre Normatif Du système MED-ÉCHO.

[40] Ministère de la Santé et des Services sociaux (MSSS). Cadre normatif du Système d’information sur la clientèle et les services des CLSC (I-CLSC) Québec: Direction genérale des ainés et des proches aidants. Ministère de la Santé et des Services sociaux; 2022.

[41] Baker F, Intagliata J (1982). Quality of life in the evaluation of community support systems. Eval Program Plan.

[42] Caron J, Mercier C, Tempier R (1997). [Validation of satisfaction with Life Domains Scale in Quebec]. Sante Ment Que.

[43] Statistics Canada (2002). Canadian Community Health Survey (CCHS).

[44] Frank C, Zamorski MA, Colman I (2018). Stigma doesn’t discriminate: physical and mental health and stigma in Canadian military personnel and Canadian civilians. BMC Psychol.

[45] Hunter BA, Mohatt NV, Prince DM, Thompson AB, Matlin SL, Tebes JK (2017). Socio-psychological mediators of the relationship between behavioral health stigma and psychiatric symptoms. Soc Sci Med.

[46] Huynh C, Kisely S, Rochette L, Pelletier E, Jutras-Aswad D, Larocque A, Fleury MJ, Lesage A. Using administrative health data to estimate prevalence and mortality rates of alcohol and other substance-related disorders for surveillance purposes. Drug Alcohol Rev; 2021. 10.1111/dar.13235 33432695

[47] Bohn MJ, Babor TF, Kranzler HR (1995). The Alcohol Use Disorders Identification Test (AUDIT): validation of a screening instrument for use in medical settings. J Stud Alcohol.

[48] Skinner HA (1982). The drug abuse screening test. Addict Behav.

[49] Simard M, Sirois C, Candas B (2018). Validation of the combined Comorbidity Index of Charlson and Elixhauser to predict 30-Day mortality across ICD-9 and ICD-10. Med Care.

[50] Association des médecins psychiatres du Québec (AMPQ). Bienvenue sur le site de l’Association des médecins psychiatres du Québec. Site web: https://ampq.org/ (consulted October 5, 2023).

[51] Stergiopoulos V, Gozdzik A, Cohen A, Guimond T, Hwang SW, Kurdyak P, Leszcz M, Wasylenki D (2017). The effect of brief case management on emergency department use of frequent users in mental health: findings of a randomized controlled trial. PLoS ONE.

[52] Chiu YM, Vanasse A, Courteau J, Chouinard MC, Dubois MF, Dubuc N, Elazhary N, Dufour I, Hudon C (2020). Persistent frequent emergency department users with chronic conditions: a population-based cohort study. PLoS ONE.

[53] Fleury MJ, Fortin M, Rochette L, Grenier G, Huỳnh C, Pelletier E, Vasiliadis HM (2019). Assessing quality indicators related to mental health emergency room utilization. BMC Emerg Med.

[54] Beck A, Sanchez-Walker E, Evans LJ, Harris V, Pegler R, Cross S (2016). Characteristics of people who rapidly and frequently reattend the emergency department for mental health needs. Eur J Emerg Med.

[55] Enders CK (2003). Using the expectation maximization algorithm to estimate coefficient alpha for scales with item-level missing data. Psychol Methods.

[56] Ali BB, Massmoudi Y. K-means clustering based on gower similarity coefficient: A comparative study. In *5th International Conference on Modeling, Simulation and Applied Optimization (ICMSAO)*. 2013.

[57] Everitt BS, Landaum S, Leese M, Stahl D (2011). Cluster Analyis.

[58] StataCorp (2021). Stata Statistical Software: Release 17.

[59] Bombak AE (2013). Self-rated health and public health: a critical perspective. Front Public Health.

[60] Berghofer A, Martin L, Hense S, Weinmann S, Roll S (2020). Quality of life in patients with severe mental Illness: a cross-sectional survey in an integrated outpatient health care model. Qual Life Res.

[61] Caron J, Tempier R, Mercier C, Leouffre P (1998). Components of social support and quality of life in severely mentally ill, low income individuals and a general population group. Community Ment Health J.

[62] IsHak WW, Elbau I, Ismail A, Delaloye S, Ha K, Bolotaulo NI, Nashawati R, Cassmassi B, Wang C (2013). Quality of life in borderline personality disorder. Harv Rev Psychiatry.

[63] Luciano A, Bond GR, Drake RE (2014). Does employment alter the course and outcome of schizophrenia and other severe mental illnesses? A systematic review of longitudinal research. Schizophr Res.

[64] Bouwmans C, de Sonneville C, Mulder CL, Hakkaart-van Roijen L (2015). Employment and the associated impact on quality of life in people diagnosed with schizophrenia. Neuropsychiatr Dis Treat.

[65] Richter D, Hoffmann H (2017). Preference for Independent Housing of Persons with Mental disorders: systematic review and Meta-analysis. Adm Policy Ment Health.

[66] Thomson RM, Igelstrom E, Purba AK, Shimonovich M, Thomson H, McCartney G, Reeves A, Leyland A, Pearce A, Katikireddi SV (2022). How do income changes impact on mental health and wellbeing for working-age adults? A systematic review and meta-analysis. Lancet Public Health.

[67] Combalbert N, Ferrand C, Pennequin V, Keita M, Geffray B (2017). Mental disorders, perceived health and quality of life of older prisoners in France. Geriatr Psychol Neuropsychiatr Vieil.

[68] Muller AE, Skurtveit S, Clausen T (2016). Many correlates of poor quality of life among substance users entering treatment are not addiction-specific. Health Qual Life Outcomes.

[69] Henkel D (2011). Unemployment and substance use: a review of the literature (1990–2010). Curr Drug Abuse Rev.

[70] Cranford JA (2014). DSM-IV alcohol dependence and marital dissolution: evidence from the national epidemiologic survey on Alcohol and related conditions. J Stud Alcohol Drugs.

[71] Kingston REF, Marel C, Mills KL. A systematic review of the prevalence of comorbid mental health disorders in people presenting for substance use treatment in Australia. Drug Alcohol Rev 2017, 36:527–39. 10.1111/dar.12448 27786426

[72] McHugh RK, Votaw VR, Sugarman DE, Greenfield SF (2018). Sex and gender differences in substance use disorders. Clin Psychol Rev.

[73] Fleury M-J, Fortin M, Rochette L, Lesage A, Vasiliadis H-M, Huỳn C, Grenier G, Pelletier E (2019). Surveillance De L’utilisation Des urgences Au Québec par les patients ayant des troubles mentaux.

[74] Wu LT, Swartz MS, Wu Z, Mannelli P, Yang C, Blazer DG (2012). Alcohol and Drug Use Disorders among adults in emergency department settings in the United States. Ann Emerg Med.

[75] Huynh C, Tremblay J, Fleury MJ (2016). Typologies of individuals attending an addiction Rehabilitation Center based on diagnosis of Mental disorders. J Subst Abuse Treat.

[76] Javaras KN, Zanarini MC, Hudson JI, Greenfield SF, Gunderson JG (2017). Functional outcomes in community-based adults with borderline personality disorder. J Psychiatr Res.

[77] Zanarini MC, Frankenburg FR, Reich DB, Fitzmaurice G (2010). The 10-year course of psychosocial functioning among patients with borderline personality disorder and axis II comparison subjects. Acta Psychiatr Scand.

[78] IsHak WW, Greenberg JM, Balayan K, Kapitanski N, Jeffrey J, Fathy H, Fakhry H, Rapaport MH (2011). Quality of life: the ultimate outcome measure of interventions in major depressive disorder. Harv Rev Psychiatry.

[79] Grambal A, Prasko J, Kamaradova D, Latalova K, Holubova M, Sedlackova Z, Hruby R (2016). Quality of life in borderline patients comorbid with anxiety spectrum disorders - a cross-sectional study. Patient Prefer Adherence.

[80] Cailhol L, Jeannot M, Rodgers R, Guelfi JD, Perez-Diaz F, Pham-Scottez A, Corcos M, Speranza M (2013). Borderline personality disorder and mental healthcare service use among adolescents. J Pers Disord.

[81] Sharp C, Green KL, Yaroslavsky I, Venta A, Zanarini MC, Pettit J (2012). The incremental validity of borderline personality disorder relative to major depressive disorder for suicidal ideation and deliberate self-harm in adolescents. J Pers Disord.

[82] Grendas L, Rodante D, Rojas S, Puppo S, Vidjen P, Lado G, Portela A, Daray FM (2017). Determinants of mental and physical health-related quality of life among patients hospitalized for suicidal behavior. Psychiatry Res.

[83] Besch V, Magnin C, Greiner C, Prada P, Debbane M, Poulet E (2023). Examining readmission factors in psychiatric emergency care for individuals with personality disorders: a 6-year retrospective study. Personal Disord.

[84] Carli P, Télion C. 20 mois de COVID-19 et après? J Eur Des Urgences et de Reanim 2021, 33:194–7.

