# Supplementary material for: Profiles of quality of life among patients using emergency departments for mental health reasons
Source: Health Qual Life Outcomes. 2023 Oct 26;21:116. doi: 10.1186/s12955-023-02200-3 (PMC10601205; doi:10.1186/s12955-023-02200-3)
Supplement: Supplementary file 1 — Supplementary Material 1 [file 12955_2023_2200_MOESM1_ESM.docx]

**Appendix 1: Codes for mental disorders, including substance-related disorders and chronic physical illnesses, according to the International Classification of Diseases, Tenth revision**

| **Diagnoses** | ***International Classification of Diseases, Tenth Revision, Canada* (*ICD-10-CA)*** |
| --- | --- |
| **Mental disorders (MD)** ^a^ | |
| ***Serious MD*** | |
| Schizophrenia spectrum and other psychotic disorders | F20* (schizophrenic disorders); F22* (persistent delusional disorders); F23 (acute and transient psychotic disorders); F24* (induced delusional disorder); F25* (schizoaffective disorders); F28* (other psychotic disorder not due to a substance or known physiological condition); F29* (unspecified psychosis not due to a substance or known physiological condition); F448 (other dissociative and conversion disorders); F481 (depersonalization - derealization syndrome) |
| Bipolar disorders | F300-F302, F308, F309 (manic episode); F310-F317, F318, 319 (bipolar episode) |
| ***Personality disorders*** | F600 (paranoid personality disorder); F61 (mixed and other personality disorders); F340 (cyclothymic disorder); F341 (dysthymic disorder); F601 (schizoid personality); F603 (borderline personality disorder); F605 (obsessive-compulsive personality disorder); F604 (histrionic personality disorder); F607 (dependent personality disorder); F602 (antisocial personality disorder); F609 (unspecified personality disorder); F21 (schizotypal personality); F606 (avoidant personality disorder); F608 (other specified personality disorders); F681 (factitious disorder); F688 (other specified disorders of adult personality and behavior); F69 (unspecified disorder of adult personality and behavior) |
| ***Common MD*** | |
| Depressive disorders | F320- F323 (major depressive disorder, single episode); F328 (other depressive episodes); F329 (depressive episode, unspecified); F330-F334 (major depressive disorder, recurrent); F338 (other recurrent depressive disorders); F339 (recurrent depressive disorder, unspecified); F348 (other persistent mood [affective] disorders); F380, F381 (persistent mood [affective] disorder, unspecified); F388 (other specified mood [affective] disorders); F39 (unspecified mood [affective] disorders); F412* (mixed anxiety and depressive disorder)* |
| Anxiety disorders | F40 (phobic anxiety disorders); F41(other anxiety disorders); F42 (obsessive-compulsive disorder); F45 (somatoform disorders); F48 (other neurotic disorders); F93, F94 (disturbance of emotions specific to childhood and adolescence) |
| Adjustment disorders | F430 (acute stress reaction); F431 (post-traumatic stress disorder); F432 (adjustment disorders); F438 (other reactions to severe stress); F439 (reaction to severe stress, unspecified) |
| Attention deficit/hyperactivity disorder | F900; F901; F908; F909 (attention deficit/hyperactivity disorder) |
| **Suicide attempt** ^a, b^ | X60-Y09, Y870, Y871, Y35-Y36, Y890, Y891 |
| **Substance-related disorders (SRD)** ^a^ |  |
| Alcohol-related disorders | F101*, F102* (alcohol abuse or dependence); F103, F104* (alcohol withdrawal); F105-F109, K700*-K704*, K709*, G621*, I426, K292*, K852, K860, E244, G312, G721, O354 (alcohol-induced disorders); F100*, T510, T511*, T518, T519 (alcohol intoxication) |
| Cannabis-related disorder | F121, F122 (cannabis abuse or dependence); F123-F129 (cannabis-induced disorders); F120, T407 (cannabis intoxication) |
| Drug-related disorders other than cannabis | F111, F131, F141, F151, F161, F181, F191, F112, F132, F142, F152, F162, F182, F192 (drug abuse or dependence); F113-F114, F133-F134, F143-F144, F153-F154, F163-F164, F183-F184, F193-F194 (drug withdrawal) F115-F119, F135-F139, F145-F149, F155-F159, F165-F169, F185-F189, F195-F199 (drug-induced disorders); F110, F130, F140, F150, F160, F180, F190, T400-T406, T408, T409, T423, T424, T426, T427, T435, T436, T438, T439, T509, T528, T529 (drug intoxication) |
| **Chronic physical illnesses** ^a,^ ^c^ | |
| Renal failure | I120, I131, N18, N19, N250, Z49, Z940, Z992 |
| Cerebrovascular illnesses | G45, G46, I60-I69 |
| Neurological illnesses | G10–G12, G13, G20, G21–G22, G254, G255, G312, G318, G319, G32, G35, G36, G37, G40, G41, G931, G934, R470, R56 |
| Endocrine illnesses (hypothyroidism; fluid electrolyte disorders and obesity) | E00, E01, E02, E03, E890; E222, E86, E87; E66 |
| Any tumor with or without metastasis (solid tumor without metastasis; lymphoma) | C00–C26, C30–C34, C37–C41, C43, C45-C58, C60–C76, C77-C79, C80; C81-C85, C88, C900, C902, C96 |
| Chronic pulmonary illnesses | I278, I279, J40-J47, J60-J64, J65, J66, J67, J684, J701, J703 |
| Diabetes complicated and uncomplicated | E102-E108, E112-E118, E132-E138, E142-E148; E100, E101, E109, E110, E111, E119, E130, E131, E139, E140, E141, E149 |
| Cardiovascular illnesses (congestive heart failure; cardiac arrhythmias; valvular illnesses; peripheral vascular illnesses; myocardial infarction; hypertension and pulmonary circulation illnesses) | I099, I110, I130, I132, I255, I420, I425–I429, I43, I50, P290; I441–I443, I456, I459, I47–I49, R000, R001, R008, T821, Z450, Z950; A520, I70-I72, I730, I731, I738, I739, I771, I790, K551, K558, K559, Z958, Z959; I05–I08, I091, I098, I34–I39, Q230–Q233, Q238, Q239, Z952, Z953, Z954I210-I214, I219, I220, I221, I228, I229, I252; I101, I100, I11, I1500, I1501, I1510, I1511, I1521, I1581, I1590, I1591, I674; I26, I27, I280, I288, I289 |
| Other chronic physical illness categories (blood loss anemia; ulcer illnesses; liver illnesses; AIDS/HIV; rheumatoid arthritis/collagen vascular illnesses, coagulopathy; weight loss, paralysis; deficiency anemia) | D500; K257, K259, K267, K269, K277, K279, K287, K289; B20-B24; D65–D68, D691, D693-D696; B18, I85, I864, I982, K700- K703, K709 K711, K713–K715, K716, K717, K721, K729, K73, K74, K754, K760, K761, K763, K764, K765, K766, K768, K769, Z944; L900, L940, L941, L943, M05, M06, M08, M120, M123, M30, M31, M32–M35, M45, M460, M461, M468, M469; G041, G114, G80, G81, G82, G83; E40–E46, R634, R64, D51–D53, D63, D649; D501, D508; D509 |

^a^ The Canadian Tenth Revision (ICD-10-CA) was used in MED-ECHO (*Maintenance et exploitation des données pour l’étude de la clientèle hospitalière*, hospitalization database) and in BDCU (*Banque de données communes des urgences*, emergency department [ED] database). Diagnoses related to the two databases were considered, and all data were integrated each year, for each patient. ^b^ Diagnostic codes for suicide attempt were registered in the MED-ECHO database. ED use for reasons of suicide ideation or attempt (suicidal behaviors) were reported by triage nurses in ED and registered in the BDCU database. As suicidal behaviors are not diagnostic codes, they were not reported in this table. ^c^ The list of chronic physical illnesses is based on an adapted and validated version of the Elixhauser Comorbidity Index, integrating the Charlson Index, which consists of 32 major categories of physical illnesses (see reference in the Methods section). For this list of chronic physical illnesses, three categories of MD and two categories of SRD (identified with an asterisk [*]) were also included under MD-SRD, thus appearing twice.
